# Defining the bellwether procedures and processes for global trauma care: an international Delphi study

**DOI:** 10.1136/bmjgh-2025-020909

**Published:** 2026-02-20

**Authors:** Michael F Bath, Joachim Amoako, Thomas Edmiston, Amila Sanjiva Ratnayake, Daphne Kabatoro, Dinesh Bagaria, Raj Menon, Jared M Wohlgemut, Laura Hobbs, Brandon G Smith, Carlos M Nuño-Guzmán, Sebastian E Vélez, Rick Brennan, Andrew Conway Morris, Timothy Craig Hardcastle, Thomas Weiser, Tom Bashford, on behalf of the Global Trauma Care Delphi Collaborative, Aarne Feldheiser

**Affiliations:** 1 International Health Systems Group, Department of Engineering, University of Cambridge, Cambridge, UK; 2 University of Ghana Medical School, Accra, Ghana; 3 Department of Surgery, Korle Bu Teaching Hospital, Accra, Ghana; 4 Department of Surgery, Army Hospital, Colombo, Sri Lanka; 5 Department of Anaesthesia, Kampala Hospital, Kampala, Uganda; 6 Department of Surgery, All India Institute of Medical Sciences, New Delhi, Delhi, India; 7 Department of Surgery, National University Hospital, Singapore; 8 Department of General Surgery, Royal Alexandra Hospital, Paisley, UK; 9 Centre for Trauma Sciences, Queen Mary University of London, London, UK; 10 NIHR Global Health Research Group on Acquired Brain and Spine Injury, University of Cambridge, Cambridge, UK; 11 Department of Anaesthesia, East & North Hertfordshire NHS Trust, Stevanage, UK; 12 Hospital Civil de Guadalajara Fray Antonio Alcalde, Guadalajara, Mexico; 13 Centro Universitario de Ciencias de la Salud, Universidad de Guadalajara, Guadalajara, Mexico; 14 Hospital de Urgencias de Córdoba, Córdoba, Argentina; 15 Global Health Consultant, -, Sydney, New South Wales, Australia; 16 JVF Intensive Care Unit, Addenbrooke’s Hospital, Cambridge University Hospitals NHS Foundation Trust, Cambridge, UK; 17 Perioperative, Acute, Critical Care and Emergency Medicine Section, Department of Medicine, University of Cambridge, Cambridge, UK; 18 Department of Surgical Sciences, Nelson R Mandela School of Clinical Medicine, University of KwaZulu-Natal College of Health Sciences, Durban, South Africa; 19 Trauma and Burns Unit, Inkosi Albert Luthuli Central Hospital, KwaZulu-Natal Department of Health, Durban, South Africa; 20 Department of Surgery, Stanford University, Stanford, California, USA; 21 Department of Anaesthesia, Cambridge University Hospitals NHS Foundation Trust, Cambridge, UK

**Keywords:** Delivery of Health Care, Health policy, Health systems evaluation, Traumatology

## Abstract

**Background:**

The complexity of delivering trauma care makes the assessment of its provision challenging. The identification of bellwether procedures has previously been successful in the evaluation of global surgical care; however, any equivalent in assessing trauma care is currently lacking. Through a Delphi process, we aimed to produce the bellwether procedures and processes for global trauma care.

**Methods:**

A global Delphi process was undertaken with healthcare professionals and academics involved in trauma care from across the world. A list of potential procedures and processes was identified through literature review and expert opinion, along with subsequent additional options suggested by respondents. Three successive rounds were completed, with respondents rating the importance of each procedure or process to be undertaken at any hospital that cares for trauma patients using a five-point Likert scale.

**Results:**

A total of 411 respondents from 78 countries completed the initial round of the Delphi process, with minimal attrition observed across rounds. Following three successive rounds of the Delphi and functional aggregation, nine bellwethers of global trauma care were determined, subdivided into three functional categories: ‘Resuscitation & Stabilisation’—(1) Advanced Airway Management, (2) Short-term C-spine Immobilisation, (3) Long Bone Immobilisation; ‘Diagnosis & Monitoring’—(4) Blood Gas Analysis, (5) Focused Assessment with Sonography in Trauma (FAST) Scanning, (6) Continuous Access to CT Imaging; ‘Optimisation & Intervention’—(7) Blood Transfusion, (8) Tube Thoracostomy, (9) Laparotomy and Splenectomy.

**Conclusion:**

The Global Trauma Care Delphi study has produced nine metrics that provide pragmatic indicators for the overall assessment of trauma care capabilities at any healthcare setting worldwide. These bellwethers of global trauma care can enable hospitals, local managers and health ministries to identify institutions or regions that may require more in-depth assessment, allowing standards in the management of traumatic injuries to improve.

**WHAT IS ALREADY KNOWN ON THIS TOPIC**

- The delivery of trauma care is complex, which makes the appropriate assessment of its provision challenging.
- Bellwether procedures for global surgery have previously been produced; however, no similar indicators for global trauma care have been successfully implemented.

**WHAT THIS STUDY ADDS**

- Through an international Delphi process of trauma healthcare professionals and academics, we have produced a consensus of nine bellwether procedures and processes for global trauma care.

**HOW THIS STUDY MIGHT AFFECT RESEARCH, PRACTICE OR POLICY**

- The bellwethers produced can act as indicators for the overall provision of trauma care for any hospital or region worldwide.
- These will allow institutions, local managers and health ministries the ability to identify areas or regions that may require further assessment and strengthening.

## Introduction

 Over 5 billion people worldwide are unable to access safe surgical care.^1^ Trauma is not excluded from this, with traumatic injuries accounting for around one in ten of all global deaths^2^ and resulting in the largest loss of disability-adjusted life years of any pathology for individuals of working age.^3^ Moreover, access is often worse in lower-income settings,^4^ where nine out of ten people cannot access basic surgical care.^1^ Adequate trauma care is fundamental to any functioning healthcare system, not just in improving mortality outcomes,^5 6^ but also in limiting the subsequent impact on disability and economic productivity.^7^ Indeed, the publication of the World Bank’s most recent Disease Control Priorities identified 43 essential surgical procedures necessary for all health systems globally, of which around a quarter were trauma-related.^8^

Ensuring comprehensive trauma care requires a complex system of intersecting processes and behaviours, all linking across co-existing healthcare services and regional infrastructure.^9^ These component parts are often interdependent, meaning that improvements in only one area may not be reflected in the overall outcome metrics. The measurement of the effectiveness of a health system is key to accountability and improvement,^10^ yet current initiatives to benchmark quality of care across global trauma systems remain inadequate. The measurement of the quality of a health system as a whole is essential yet rarely done; current quality measurements of health systems are typically divided by disease, focused on inputs rather than outcomes, or poorly aligned to population health needs.^10^ Resolutions from the World Health Assembly have previously highlighted the cost-effectiveness that optimal trauma care can offer to a region and emphasise the need for improved organisation in such care.^11^ The development of nuanced and focused measures of trauma care worldwide is therefore a must.

The bellwether procedures of global surgery, comprising Caesarean delivery, laparotomy and treatment of an open fracture, have been proposed as markers of an effective surgical system and are closely associated with the ability to perform all obstetric, general, emergency and orthopaedic procedures in low- and middle-income countries.^12^ These bellwethers have been recommended to act as pragmatic markers of wider surgical procedures, while similar work in elective surgical health systems has suggested the use of a ‘tracer condition’ to map clinical effectiveness.^13^ However, trauma-related injuries have a distinct epidemiological pattern compared with general surgery cases, with an added complexity of time dependency.^14^ To date, no bellwether procedures or processes have been defined for trauma specifically, limiting the ability to benchmark different trauma systems across different contexts.^15^

The aim of this Delphi exercise was to determine the bellwether procedures or processes of global trauma care, as a proxy measurement of trauma care effectiveness and quality.

## Methodology

The Global Trauma Care Delphi Study was conducted in accordance with a pre-published and publicly available study protocol.^16^ The study was designed and conducted by an international steering committee consisting of expert clinical academic representatives working across multiple economic and geographic settings, identified through research links, clinical networks and existing collaborations. The study has been reported following Conducting and Reporting Delphi Studies guidelines.^17^

Major trauma was defined as a ‘significant injury or injuries that have potential to be life-threatening or life-changing sustained from either high energy mechanisms or low energy mechanisms in those rendered vulnerable by extremes of age’.^18^ This broad definition of major trauma allowed for the focus to shift from a purely mortality-focused trauma outcome to a more holistic definition that takes account of morbidity and rehabilitation potential.^7^

Any healthcare professional involved in trauma care from any country globally was eligible to be involved. Participants were identified through a purposive snowballing technique, using pre-existing collaborative networks, communication channels and research partners. Given the broad perspectives required to answer the research question, a high heterogeneity in the breadth of trauma care experience among the respondents was required, therefore a planned sample size of 400 respondents for the first round was required, from across all relevant sub-specialities involved in trauma care.

### Delphi design

Three sequential rounds of the Delphi process were undertaken between October 2024 and March 2025. Given the multi-national location of respondents, it was conducted using the secure online platform Qualtrics XM (Qualtrics, Provo, USA). An initial draft list of potential procedures and processes for the first round of the Delphi was compiled through review of the wider literature^8 12 19 20^ and then revised through expert opinion from the group’s steering committee, all of whom had expertise in trauma care across multiple geographic and economic settings (online supplemental material). Appropriate definitions for each option included were provided where necessary.

In each round, participants were asked to rank each measure using a five-point Likert scale, with measures that obtained consensus proceeding to the next round. Given the relative heterogeneity of the participants, consensus for a given statement in each round required a median score of >3.5, with the additional criterion of the IQR of ≤1 from round 2 onwards and showing stability across rounds.^21^ In cases of duplicate entries by a respondent in a round, only the first response was included and any subsequent entries excluded. Data were summarised using median and IQR for ordinal data, and number and percentage for categorical data.

During Round 1, respondents were also given a free text option to submit any additional options for consideration in subsequent rounds of the Delphi exercise, with options that were suggested by multiple respondents across all contexts included. Following additional feedback available in Round 1, further adjustments were also made to the wording of the options that had reached consensus, to ensure clarity and language consistency (online supplemental material).

Following the Delphi process, a method of functional aggregation was performed by members of the steering committee, whereby the final procedures and processes were aggregated by their function(s) and those options which represented the widest spread of functionalities were included. This ensured that the overall function of the selected procedures and processes remained, while also minimising the number of included metrics.

### Patient and public involvement

A series of patient and public involvement and engagement (PPIE) focus groups were held in both the UK and Uganda. These discussions sought to elicit stakeholder perspectives on the study’s overarching rationale and the anticipated outcomes of the Delphi process. By engaging participants from two markedly different sociocultural contexts, the focus groups aimed to capture lay perspectives and understanding of the relevance, clarity and applicability of the developing findings and proposed recommendations. This step was imperative in ensuring that the final outputs of the process would be not only methodologically sound, but also meaningful and implementable across a range of settings and contexts, particularly with respect to their acceptability, feasibility and cultural sensitivity.

Overall, in both settings, attendees found the concept of the study understandable and beneficial to improving global trauma standards. In the UK, questions were raised about other standards or measures that could be used, which led to further discussions regarding the need for comparable measurements that can be easily measured globally. In Uganda, it was discussed whether such work could eventually inform a triaging system for the public when attempting to access care following injury and potential solutions to this.

## Results

**Figure 1:**
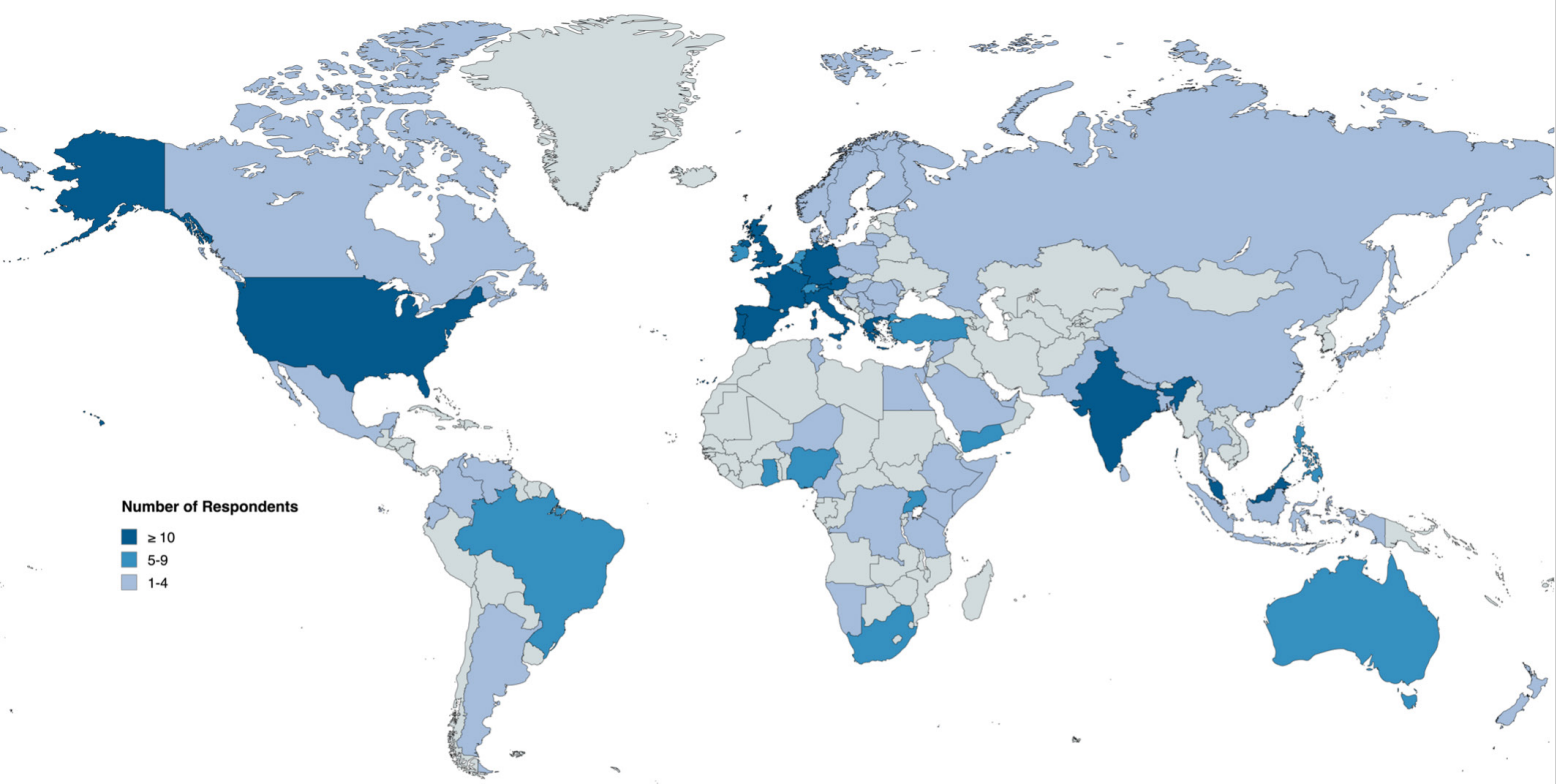
Map demonstrating the global distribution of responding collaborators for Round 1 (created with mapchart.net)

The first round of the Delphi process had 411 respondents (table 1) from across 78 countries (figure 1), covering all six inhabited continents and all four Human Development Index (HDI) levels (online supplemental material). The majority were medical doctors (396 respondents, 96.3%), with the most common specialities reported as Critical Care (199 respondents, 48.4%), Surgery (109 respondents, 26.5%), Anaesthesia (70 respondents, 17.0%) and Pre-Hospital or Emergency Medicine (26 respondents, 6.3%) (table 1). Of those in surgical specialties, the most common sub-specialties were General Surgery (52 respondents, 47.7%) and Trauma Surgery (30 respondents, 27.5%).

**Table 1:** Characteristics of Respondents, n=411

Characteristic	Respondent numbers
Profession	Doctor—396 (96.4%)
	Nurse—8 (1.9%)
	Other—7 (1.7%)
Specialty	Critical care—199 (48.4%)
	Surgery—109 (26.5%)
	Anaesthesia—70 (17.0%)
	Pre-hospital or emergency medicine—26 (6.3%)
	Other—5 (1.2%)
Surgical sub-specialty (n=109)	Trauma surgery—30 (27.5%)
	General surgery—52 (47.7%)
	Orthopaedic surgery—6 (5.5%)
	Neurosurgery—14 (12.8%)
	Paediatric surgery—2 (1.8%)
	Vascular surgery—1 (0.9%)
	Plastic surgery—5 (4.6%)

Attrition between Round 1 and Round 2=75.9%; attrition between Round 2 and Round 3=92.0%.

Retention of respondents between the first and second round was 75.9%, and between the second and third round was 92.0%. Between rounds 1 and 3, the distribution of professions, specialities and sub-specialities remained consistent despite attrition. Similarly, the most-represented countries remained largely consistent across all three rounds, with no significant change in the balance of HDI representation.

After the first round of the Delphi process, from the initial 32 procedures or processes listed, 7 were removed due to a lack of consensus. Free text responses led to the subsequent addition of four new options and edits to three original options (online supplemental material). After the second round of the Delphi, a further 18 options were lost, and after the final round, a further option was lost, leaving 13 options at the end of the Delphi process.

The final bellwether procedures and processes were then determined through functional aggregation. Through expert review, the functional ability of each selected option from the Delphi process was determined, and any option where this ability was presupposed by another option within the system was removed. For example, ‘laparotomy and splenectomy’ was deemed to predispose ‘laparotomy and packing’, and therefore they were able to be amalgamated. This produced a final list of nine procedures and processes as the bellwethers of global trauma care (table 2).

**Table 2:** The final bellwether procedures and processes for global trauma care

Resuscitation and stabilisation	Diagnosis and monitoring	Optimisation and intervention
Advanced airway management	Blood gas analysis	Blood transfusion
Short-term C-spine immobilisation	FAST scanning	Tube thoracostomy
Long bone immobilisation	Continuous access to CT imaging	Laparotomy and splenectomy

CT, computed tomography; FAST, Focused Assessment with Sonography in Trauma.

**Figure 2:**
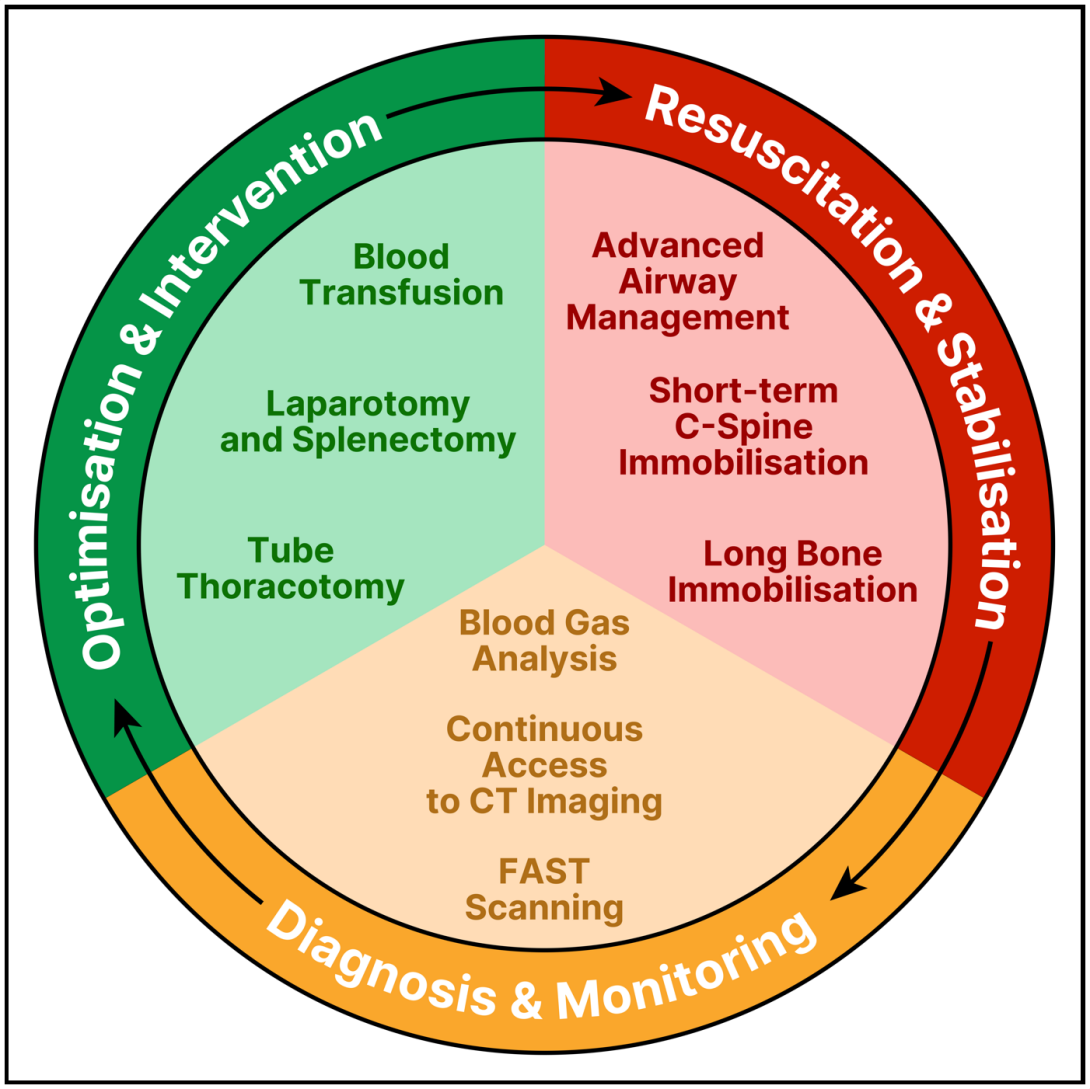
Schematic representing the nine bellwether procedures and processes of global trauma care. FAST, Focused Assessment with Sonography in Trauma.

A second functional aggregate step was performed, whereby functional subsystems of trauma care were defined based on the nine finalised bellwethers, leading to three key phases of care: Resuscitation and Stabilisation, Diagnosis and Monitoring, and Optimisation and Intervention (figure 2).

## Discussion

Through our global Delphi process, we have defined the nine bellwether procedures and processes for global trauma care. After three successive rounds, our iterative process has allowed the generation of core indicators for global trauma care, with consensus achieved from a large group of healthcare professionals and academics involved in trauma care from across the world. These metrics can enable institutions, local managers or health ministries globally to rapidly assess any hospital’s proficiency in providing trauma care and to identify institutions or regions that may require further support and targeted action. The bellwethers we propose encompass both procedures and processes that should be performed by any hospital worldwide, not just specific operations, and represent a full spectrum of trauma care warranted by a healthcare system.

Measuring the quality of a health system as a whole, across all aspects of the patient pathway, is essential yet rarely done.^10^ Trauma care is no exception to this. Concerns have previously been raised about current measures of care being too disease-specific or focused solely on inputs rather than relevant outcomes.^10^ However, our proposed bellwethers of trauma care are both wide-ranging, involving functional aspects from across the trauma care pathway, and pragmatic, to ensure an effective and widespread use as indicators of global trauma care proficiency. This combination of procedures and processes will allow for the assessment of entire healthcare systems, from district hospitals through to tertiary referral centres, and identify regional disparities within a country by assessing the presence of these bellwethers. Measuring metrics not just focused on the operating theatre setting is also key, as it is well known that the period between the point of injury to the provision of definitive care is just as important as any intra-operative intervention or rehabilitation.^22^,^25^ Select measures that would conventionally form part of trauma care checklists, such as tranexamic acid administration, have not been included in our list; however, importantly, many of these fall within the select procedures or processes listed. This adaptability and flexibility are key strengths of the bellwethers. Previously proposed capacity assessments have been limited in their use, with their focus restricted to certain settings or regions^26^ or no representation of specific trauma-related care,^27^ while also needing significant time and resources to perform. The use of these global trauma bellwethers provides an opportunity for rapid screening of a trauma service of any hospital worldwide, to identify the institutions or regions within a healthcare network where further assessment is warranted, recommendations for which have been previously described.^28^

Keeping the bellwethers of global trauma care broad allows for wider applicability in their deployment. We envisage they can be used to investigate parameters within global trauma care, from need to access to quality,^29^ and importantly can be adapted to meet local requirements or at a higher policy level, a strength demonstrated previously with the WHO safe surgery checklist^30^ or the WHO trauma care checklist.^31^ Indeed, there has been clear overlap between many of the bellwethers reported and other previous published Delphi processes on emergency management in mass casualty.^32^ Trauma care is becoming increasingly complex, with many regions’ trauma networks embedded within existing emergency services and co-ordination between pre-hospital, emergency departments, anaesthesia and intensive care, surgical teams and rehabilitation.^33^ This complexity leads to challenges when attempting to assess and monitor trauma services; despite the efforts of agencies, such as the WHO through their International Registry for Trauma and Emergency Care and regional trauma initiatives,^34^ it remains challenging to understand existing trauma system performance, compare systems with other settings and plan targeted interventions to improve outcomes. In systems engineering, indicators are widely used as measures that ‘provide the insight needed to identify opportunities for improvement’^35^ and this cross-speciality application of these bellwethers as indicators of trauma care functioning can ensure the most effective means of identifying and comparing hospitals or regions that may warrant further assessment and improvement. Our PPIE work also highlighted the potential this work could have in use by the public to triage themselves to an appropriate level hospital after injury - more work on this topic is now required to ensure the optimal implementation of these bellwethers.

We used functional aggregation to allow for a focused set of indicators to be produced, while not losing the spectrum of care covered. The simplicity in the previous bellwethers of global surgery, through utilisation of only three metrics, has been, in part, a significant contribution to their success; they have been used by multiple countries across a range of resource settings,^36^,^40^ alongside their incorporation into the Lancet Global Surgery commission,^1^ while other such tools containing a higher number of metrics have had a more limited uptake.^20^ We envisage that the select number of procedures and processes chosen in our bellwethers of global trauma care, that represent the core functional headings of the trauma care pathway, will allow for their widespread uptake and use. Importantly, we have not defined where geographically these procedures or processes should be performed, as trauma care can be perceived to start from the time of injury in the community through to rehabilitation back in the community, not limited solely to the hospital setting; as such, the way in which these bellwethers are used can be adapted to local context and structure. Framing these bellwethers as functional capabilities, rather than linking them to any specific equipment or infrastructure requirements, allows them to remain contextually agnostic and ensure their global relevance. This work further coincides with the 76th World Health Assembly call for integrated emergency, critical and operative care^41^ and has the potential to be incorporated into country-level packages or policy when attempting to implement universal health coverage agendas.

This study comes with certain limitations. While we have conducted a global Delphi process across a wide range of healthcare professionals involved in trauma, our proposed bellwethers have not been validated across larger data sets and further work is required to demonstrate their success and applicability. However, Delphi processes have previously been used to good effect within both trauma care and global surgery^42 43^; therefore, this should not preclude any implementation in their use. Certain globally-recognised core clinical domains were not directly represented, having not reached consensus through the Delphi, such as traumatic brain injury^44 45^ or exsanguinating haemorrhage.^46^ However, rather than representing an omission or lack of appreciation by either the study team or the participants, these should be viewed as crucial findings worthy of further exploration. Finally, despite a wide range of specialties included, through our recruitment methodology, the majority of our respondents were medical doctors across a skewed distribution of represented specialties, with only a few allied healthcare professionals participating, leading to a potential selection bias and may reflect the lack of post-operative care included in the final bellwethers selected; ensuring a wider remit of healthcare professionals in any similar work must be viewed as a priority.

## Conclusion

We have conducted a large international Delphi process to produce the bellwether procedures and processes for global trauma care. The Global Trauma Care Delphi study has produced nine metrics that provide pragmatic indicators for the overall assessment of trauma care capabilities in any healthcare setting worldwide, allowing hospitals, local managers and health ministries the ability to identify institutions or regions that may require further assessment and strengthening. These bellwethers build on the success of previous markers of global surgical care, and further work is needed to demonstrate their applicability and use across a wide range of settings.

## Supplementary material

10.1136/bmjgh-2025-020909 online supplemental file 1

10.1136/bmjgh-2025-020909 online supplemental file 2

## Data Availability

All data relevant to the study are included in the article or uploaded as supplementary information.
