# Supplementary material for: Defining the bellwether procedures and processes for global trauma care: an international Delphi study
Source: BMJ Glob Health. 2026 Feb 20;11(2):e020909. doi: 10.1136/bmjgh-2025-020909 (PMC12927294; doi:10.1136/bmjgh-2025-020909)
Supplement: online supplemental file 1 [file bmjgh-11-2-s001.docx]

# Supplementary Material

1 – List of collaborators

2 – List of countries

3 – Selection process of each Delphi round

4 – CREDES checklist

Figure A1 – Flowchart of Delphi Stages

## List of Collaborators

**Writing Group**

MF Bath, J Amoako, T Edmiston, A Ratnayake, D Karusoke, D Bagaria, R Menon, JM Wohlgemut, L Hobbs, BG Smith, CM Nuño-Guzmán, SE Vélez, R Brennan, TC Hardcastle, A Conway Morris, T Weiser, T Bashford

**Protocol Development Group**

MF Bath, J Amoako, T Edmiston, A Ratnayake, D Bagaria, R Menon, JM Wohlgemut, M McKenna, K Bateman, K Hancorn, J Shepherd, S Yoong, L Hobbs, BG Smith, C Whiffin, TC Hardcastle, A Conway Morris, T Weiser, T Bashford

**Collaborators**

Aarne Feldheiser, Abeer Noureldin, Abhinav Gupta, Agnes S. Meidert, Aitor Landaluce-Olavarria, Alberto Cucino, Alessandro Strumia, Ali Guner, Amila S. Ratnayake, Ana S. Lopes, Anbin Naidoo, Andee Dzulkarnaen Zakaria, Andrea Carsetti, Andrea Gutiérrez, Andrea Sanna, Andreas Hartjes, Andres M. Rubiano, Andrew Conway Morris, Andrew G. Robertson, Anis Hasnaoui, Ankur Gupta, Ankur Sharma, Annalisa Piccolo, Anusha Cherian, Apurb Sharma, Arnav Mahajan, Arthur James, Ary S. de Sousa, Ashish A. Bartakke, Ashraf Roshdy, Asish Kumar Panda, Asyraf Mohd Zuki, Athanasios Gargavanis, Auwal Adamu, Banu Yigit, Barbara Hallmann, Believe O. Nomayo-Oriabure, Benoit Blondeau, Bhupinder Singh, Bo E. Madsen, Boris V. Tablov, Buh F. Chu, Bulent Citgez, Carlos M. Nuño-Guzmán, Carmen Lopez Soto, Christina Alexopoulou, Christopher Terblanche, Conor S. O'Flynn, Cornelis Slagt, Cristian Deana, Cynthia A. O. Akli-Nartey, Dana R. Tomescu, David A. Leon, David Benguria Puebla, David Wall, Devorah L. Wineberg, Dhuleep Wijayatilake, Diana Adrião, Diego Pastor Marcos, Diego Visconti, Dimitrios C. Papadopoulos, Dimitrios Toumpanakis, Dinesh Kumar Bagaria, Dorothy Turitwenka, Dziwornu Kunutsor, Edward J Nevins, Elena Theophilidou, Elham Rostami, Emalee J. Burrows, Emilio Rodriguez-Ruiz, Emma Muendo Loko, Emmanuel Andzie-Mensah, Emmanuel Ikwutah, Erika Noè, Esther K. Haspels-Hogervorst, Eugenia Anabel Liger Borja, Evangelia Theodorou, Evangelos Kaimakamis, Fabio Guarracino, Fabio Sangalli, Francesca Mulazzani, François Dépret, Gary A. Bass, Gentle Shrestha, George D. Mukoro, Georgia Tsaousi, Gerard Angeles-Fite, Gerard McKnight, Gerardo M. Briones, Jr., Giacomo Calini, Giacomo E. Iapichino, Giuliano Bolondi, Giuseppe Pascarella, Gizem Kılıç Aydoğdu, Harald Willschke, Harri G. Jones, Harrison Roocroft, Hasan K. Pampal, Hatem Elkady, Helena Odenstedt Herges, Hendrik Bracht, Henry O. Abiyere, Henry O. Nnajiuba, Hynek Riha, Ibrahim A. Saidu, Ifigenia Ravani, Indiradevi Bhagavatula, Inês C. R. Gomes, Inês Carolino, Ioannis Andrianopoulos, Ioannis Pantazopoulos, Ion Chesov, Isaac Chukwu, Jan A. Graw, Jan Gunst, Jan Michael V. Yap, Jeffrey N. Wood, Jessie R. Wilburn, Jihad Mallat, Joana Osório, Joaquín C. Joaco, Johannes Ehler, John R. Prowle, John V. Taylor, Jonathon D. Taylor, José M. Alonso-Íñigo, Juan A. Llompart-Pou, Juan C. Lopez-Delgado, Kalavathy Swarna, Kamal Jayasuriya, Khuram Maqbool, Kirubel Geletu, Koen M. E. M. Reyntjens, Kristina E. Fuest, KW Karen Tsang, Larissa Dsilva, Lars-Olav Harnisch, Laura Borgstedt, Laura Galarza, Leandro B. Carvalho, Lene Russell, Leonid A. Lichman, Liam D. Quinn, Lilav Hasan, Lodovico Sartarelli, Lok Ka Cheung, Lovenish Bains, Luca Carenzo, Luigi Vetrugno, Mabel Banson, Mafalda Mourisco, Mahan Sadjadi, Mansoor N. Bangash, Marc L. C. Yang, Marcel J. H. Aries, Marcella C. A. Müller, Maria Bringas, Martin Helan, Martin W. Dünser, Mate Berczi, Matthew Woods, Melanie Premstaller, Mercedes Pilkington, Mervyn Mer, Michael El-Boghdady, Michal Kalina, Michal Kawka, Michelle S. Chew, Miguel Garcia-Marin, Miguel Prista Monteiro, Mirza Aun Muhammad Baig, Mo Al-Haddad, Mohamed Almurtada Abdelwahab, Mohamed Sheikh Hassan, Mohan Gurjar, Nan Wang, Nebiyou S. Bayleyegn, Nebojsa Brezic, Neeraj Kumar, Neeru Sahni, Nele M. Baert, Nelson Ahadi Isaya, Nichole Starr, Nicolò Marchesini, Nik Ahmad Shaiffudin, Nikolaos Koronakis, Nils I. Corson, Niraj Tyagi, Oles Yehorov, Oliver Smith, Olivier Santos, Otavio Ranzani, Otavio T. Ranzani, Paër-sélim Abback, Patrick Biston, Patrick Meybohm, Patrizio Petrone, Pedro M. G. Gomes, Pedro Povoa, Peter M. Spieth, Petra O. P. Wahjoepramono, Philip I. Bastone, Philipp Venetz, Pierre Mora, Pierre Pasquier, Prashant Nasa, Priya Baby, Puvanendiran Shanmugam, Qing Yuan Goh, Rajib Hasan, Rakesh Garg, Ram Jeevan, Randeep S. Jawa, Reesha Joshi, René R. Schmutz, Richard A. Crawford, Robert K. Parker, Roberta M. L. Roepke, Robin Manidas, Ronald R. Barbosa, Ryan Breslin, Sagarika Panda, Salvatore Lucio Cutuli, Samir Samal, Samira Akbas, Sebastián E. Vélez, Sebastian Schnaubelt, Sebastien Gaujoux, Sergey E. Katorkin, Sergio M. Navarro, Sergio Marcos Contreras, Shah M. Rahman, Sharon Einav, Shilpi Karmakar, Simant K. Jha, Slavica Kvolik, Somnath Bose, Sotiria Koutsouki, Stavros Gourgiotis, Stefan P. Wirtz, Stephanie Giaquinto, Stephanie Mifsud, Sunder Balasubramaniam, Tariq H. Khan, Tchokam Lionelle, Teresa C. Guimarães, Thangaraj Munusamy, Theodoros Aslanidis, Thierry Bège, Thomas Botrel, Thomas Clavier, Timothy C. Hardcastle, Tina Tomić Mahečić, Tomasz Jodlowski, Tomasz Torlinski, Vasileios Kaldis, Vincenzo Russotto, Wael Hassan, Waleed Bin Ghaffar Waleed, Wellingson Paiva, Xavier Chapalain, Xavier-Jean Taverna, Yashoda Khadka, Yoshiro Hayashi, Yoshiro Kobe, Yunushan F. Aydoğdu, Yuti Sheth, Yuzaidi Mohamad, Zainub Jooma, Zeliha A. Ozdemirkan, Zeljka Gavranovic, Zhongheng Zhang, Zudin A Puthucheary

## List of Countries

List of countries represented by respondents, categorised by Human Development Index (HDI) levels:

**Very High HDI** – United Kingdom, United States, Germany, France, Canada, Australia, Spain, Italy, Netherlands, Sweden, Switzerland, Belgium, Portugal, Japan, South Korea, Israel, Singapore

**High HDI** – Malaysia, Mexico, Turkey, Brazil, Greece, Argentina, Sri Lanka, Costa Rica, Romania, Bulgaria, Serbia

**Medium HDI** – India, Indonesia, Bangladesh, Nepal, Ghana, Kenya, Cameroon, Namibia, Philippines, Egypt, United Republic of Tanzania

**Low HDI** – Nigeria, Ethiopia, Sudan, Afghanistan, Yemen, Democratic Republic of the Congo, Niger, Pakistan, Somalia

## Selection Process of Each Delphi Round

| **Options** | **Round 1**  (n=411) | **Round 2**  (n=312) | **Round 3**  (n=287) |
| --- | --- | --- | --- |
| Basic Airway Management | - | 5 (0) | 5 (0) |
| Advanced Airway Management*^§^* | 5 (0) | 5 (1) | 5 (1) |
| Surgical Tracheostomy | - | 4 (2) | - |
| Tube Thoracostomy (Chest Drain) | 5 (0) | 5 (0) | 5 (0) |
| Thoracotomy And Pericardial Window | 4 (2) | 3 (2) | - |
| Limb Amputation | 4 (2) | 4 (2) |  |
| Neck Exploration | 3 (2) | - | - |
| Long Bone Fixation^*^ | 4 (2) | - | - |
| Long Bone Immobilisation | - | 5 (1) | 5 (1) |
| Long Bone Fixation (Internal and External) | - | 4 (2) | - |
| Burr Hole | 4 (2) | 4 (2) | - |
| Craniotomy | 4 (3) | 3 (2) | - |
| Blood Transfusion Service | 5 (0) | 5 (0) | 5 (1) |
| Pathology Laboratory Service | 4 (3) | 4 (3) | - |
| Dedicated Critical Care Service | 5 (1) | 4 (2) | - |
| Formal Links to Pre-Hospital Care | 5 (1) | 4 (2) | - |
| Short-Term C-Spine Immobilisation | 5 (1) | 5 (1) | 5 (0) |
| Mandibular Fracture Fixation | 2 (2) | - | - |
| Physiotherapy Service | 4 (3) | 3 (2) |  |
| Occupational Therapy Service | 3 (2) | - | - |
| Diagnostic Laparoscopy | 4 (2) | 4 (2) | - |
| Laparotomy And Splenectomy | 5 (1) | 5 (1) | 4 (1) |
| Laparotomy And Bowel Resection | 4 (2) | 4 (2) | - |
| Laparotomy And Packing | 5 (1) | 5 (1) | 5 (1) |
| Vascular Graft (Extremity) | 3 (2) | - | - |
| Blood Gas Sampling^‡^ | 5 (0) | 5 (1) | 5 (1) |
| Wound Debridement | 5 (1) | 4 (1) | 4 (2) |
| Interventional Radiological Embolisation | 3 (2) | - | - |
| Pelvic Fracture Stabilisation*^†^* | 5 (1) | - | - |
| Pelvic Fracture Fixation | - | 4 (2) | - |
| Continuous Monitoring of Vital Signs | 5 (0) | 5 (0) | 5 (0) |
| Continuous Availability CT Imaging | 5 (1) | 5 (1) | 4 (1) |
| Continuous Availability Plain Film Radiographs | 5 (0) | 5 (0) | 5 (0) |
| Soft Tissue Reconstruction | 3 (1) | - | - |
| Fasciotomy | 4 (1) | 4 (2) | - |
| Escharotomy | 4 (2) | 4 (2) | - |
| Hand Exploration | 3 (2) | - | - |
| Caesarean Section | - | 4 (2) | - |
| Focused Assessment with Sonography in Trauma | - | 5 (1) | 5 (1) |
| Intracranial Pressure Monitoring | - | 3 (2) | - |
| Psychological Support Services | - | 3 (2) | - |

*Table 1 – Options available in each round for the Global Trauma Care Delphi, showing median (IQR) for each option; ^§^Updated to “Basic airway management”, “advanced airway management”, and "surgical tracheostomy” for Round 2 onwards; *Updated to “Long Bone Immobilisation” and “Long Bone Fixation (Internal & External)” respectively for Round 2 onwards;* ‡*Updated to “Blood Gas Analysis” at Functional Aggregation; †Updated to “Pelvic Fracture Fixation” for Round 2 onwards; following feedback and consensus from collaborators after Round 1, the following additional options were added for Round 2 onwards: Caesarean section; Focused Assessment with Sonography in Trauma (FAST); Intracranial pressure (ICP) monitoring; Psychological support services*

## CREDES Checklist

| **Criterion** | **Page(s)** |
| --- | --- |
| **Rationale for the choice of the Delphi technique** |  |
| Justification | 1-2 |
| **Planning and design** |  |
| Planning and process | 3-4 |
| Definition of consensus | 3-4 |
| **Study conduct** |  |
| Informational input | 3 |
| Prevention of bias | 3 |
| Interpretation and processing of results | 4 |
| External validation | 4 |
| **Reporting** |  |
| Purpose and rationale | 1 |
| Expert panel | 3 |
| Description of the methods | 3-4 |
| Procedure | Suppl. Mat. |
| Definition and attainment of consensus | 3-4 |
| Results | 5 |
| Discussion of limitations | 8 |
| Adequacy of conclusions | 8 |
| Publication and dissemination | 6-8 |

*Table A1 – Checklist of the Conducting and Reporting Delphi Studies (CREDES) guidelines*

## Flow Chart of the Stages of the Delphi
