# Supplementary material for: Defining the bellwether procedures and processes for global trauma care: an international Delphi study
Source: BMJ Glob Health. 2026 Feb 20;11(2):e020909. doi: 10.1136/bmjgh-2025-020909 (PMC12927294; doi:10.1136/bmjgh-2025-020909)
Supplement: online supplemental file 2 [file bmjgh-11-2-s002.docx]

### BMJ Global Health Author Reflexivity Statement

Adapted from Morton, B., Vercueil, A., Masekela, R., Heinz, E., Reimer, L., Saleh, S., Kalinga, C., Seekles, M., Biccard, B., Chakaya, J., Abimbola, S., Obasi, A. and Oriyo, N. (2022), Consensus statement on measures to promote equitable authorship in the publication of research from international partnerships. Anaesthesia, 77: 264-276. <https://doi.org/10.1111/anae.15597>

| **Study conceptualisation** | |
| --- | --- |
| 1. How does this study address local research and policy priorities? | Global Delphi process, whereby the aim of the study is to determine global consensus to support future research |
| 1. How were local researchers involved in study design? | Multinational writing group and steering committee involved in conceptualisation and study design |
| **Research management** | |
| 1. How has funding been used to support the local research team(s)? | No funding was involved directly for this study |
| **Data acquisition and analysis** | |
| 1. How are research staff who conducted data collection acknowledged? | Collaborative authorship model |
| 1. How have members of the research partnership been provided with access to study data? | All authors in the writing group and steering committee had full access to all the data in the study, if requested |
| 1. How were data used to develop analytical skills within the partnership? | Not applicable, as descriptive statistics only |
| **Data interpretation** | |
| 1. How have research partners collaborated in interpreting study data? | Multinational writing group and steering committee involved throughout data analysis |
| **Drafting and revising for intellectual content** | |
| 1. How were research partners supported to develop writing skills? | Support from members of the writing group was provided to research partners when requested |
| 1. How will research products be shared to address local needs? | As a Global Delphi process, this work produces a global consensus that directly supports future research that can be adapted to match local context |
| **Authorship** | |
| 1. How is the leadership, contribution and ownership of this work by LMIC researchers recognised within the authorship? | Multinational writing group and steering committee involved throughout in study write-up and represented in writing group. Collaborative authorship model. |
| 1. How have early career researchers across the partnership been included within the authorship team? | Range of expertise and seniority involved in the writing group and steering committee |
| 1. How has gender balance been addressed within the authorship? | Mix of genders in writing group and in protocol development, as represented in the authorship. Collaborative authorship models for all respondents. |
| **Training** | |
| 1. How has the project contributed to training of LMIC researchers? | Support provided in analysis and write-up to collaborators across settings |
| **Infrastructure** | |
| 1. How has the project contributed to improvements in local infrastructure? | This work has produced a series of bellwether procedures and processes that will allow the measurement of trauma care provision at a local and regional level, identifying areas for potential improvement |
| **Governance** | |
| 1. What safeguarding procedures were used to protect local study participants and researchers? | Anonymised study respondents |
